# Cardiotoxicity: A Major Setback in Childhood Leukemia Treatment

**DOI:** 10.1155/2021/8828410

**Published:** 2021-01-06

**Authors:** Diana R. Lazăr, Anca D. Farcaş, Cristina Blag, Alexandra Neaga, Mihnea T. Zdrenghea, Călin Căinap, Florin L. Lazăr, Adrian Stef, Simona S. Căinap

**Affiliations:** ^1^Emergency County Hospital for Children, Pediatric Clinic No. 2, Department of Pediatric Cardiology, Cluj-Napoca, Romania; ^2^“Iuliu Hatieganu” University of Medicine and Pharmacy, Department No. 11, Oncology, Cluj-Napoca 400012, Romania; ^3^Emergency Clinical County Hospital, Cardiology Department, Cluj-Napoca, Romania; ^4^“Iuliu Hatieganu” University of Medicine and Pharmacy, Department No. 5, Internal Medicine, Cluj-Napoca 400012, Romania; ^5^“Iuliu Hatieganu” University of Medicine and Pharmacy, Department No. 9, Mother & Child, Cluj-Napoca 400012, Romania; ^6^Emergency County Hospital for Children, Pediatric Clinic No. 2, Department of Hemato-Oncology, Cluj-Napoca, Romania; ^7^“Prof. Dr. Ion Chiricuta” Oncology Institute, Cluj-Napoca, Romania; ^8^“Nicolae Stancioiu” Heart Institute, Cluj-Napoca, Romania; ^9^“Iuliu Hatieganu” University of Medicine and Pharmacy, Department No. 7, Surgery, Cluj-Napoca 400012, Romania

## Abstract

Ongoing research in the field of pediatric oncology has led to an increased number of childhood cancer survivors reaching adulthood. Therefore, ensuring a good quality of life for these patients has become a rising priority. Considering this, the following review focuses on summarizing the most recent research in anthracycline-induced cardiac toxicity in children treated for leukemia. For pediatric cancers, anthracyclines are one of the most used anticancer drugs, with over half of the childhood cancer survivors believed to have been exposed to them. Anthracyclines cause irreversible cardiomyocyte loss, leading to chronic, progressive heart failure. The risk of developing cardiotoxicity has been known to increase with the treatment-free interval and total cumulative dose. However, because of individual variations in anthracycline metabolism, it has recently been shown that there is no risk-free dose. Moreover, studies have shown that diagnosing anthracycline-induced cardiomyopathy in the symptomatic phase is associated with poor treatment response and prognosis. Thus, early and systematic evaluation of these patients is crucial to allow optimal therapeutic intervention. Although currently echocardiographic assessment of left ventricle ejection fraction and cardiac biomarker evaluation are being used for cardiac function monitoring in oncologic patients, there is no established follow-up and treatment protocol for these patients, and these methods are neither specific nor sensitive for identifying early cardiac dysfunction. All things considered, the need for ongoing research in the field of pediatric cardiooncology is crucial to offer these patients a chance at a good quality of life as adults.

## 1. Introduction

Recent discoveries in the field of pediatric oncology have significantly improved 5-year survival rates, from 50% in the 1970s to 80% nowadays [1–4]. On the other hand, the incidence of pediatric cancers is slowly increasing [5], most noticeable for leukemia, cancer being still one of the main causes of death by illness in childhood and adolescence [1–3]. Hematopoietic malignancies are the most common cancers in children, accounting for up to 31% of all malignancies that occur in children younger than 15 years of age [1, 3, 6]. Leukemias are more common than lymphomas; the most common is acute lymphoblastic leukemia (ALL), representing up to 25% of all childhood cancers in children under 15 years old [7]. The most important prognostic factor is the correct choice of treatment based on specific group stratification. Risk assessment takes into account many factors including leukemia subtype, age and white blood cell count at diagnosis, and also response rate to the induction treatment [7, 8]. Chemotherapy is the main treatment method used in leukemia and consists of an association of several cytotoxic agents, showing an increased efficiency of up to 85% in inducing remission [3, 6].

However, efficient, oncological treatments are often aggressive, with multiple side effects that can also occur years after treatment has ended. Considering that more survivors of childhood cancer reach adulthood, special attention has been given to the quality of life of these patients, as well as to the late-onset complications of the antineoplastic treatment [2, 3, 6]. Better knowledge and understanding of these side effects are needed to amend or even prevent some of them in the future. Cardiovascular complications are one of the main causes of morbidity and mortality in survivors of childhood cancer [9, 10]. Anthracyclines (AC) represent one of the most effective chemotherapeutic agents currently used, being simultaneously the most well known for their effects on the cardiovascular system [11]. The Childhood Cancer Survivor Study (CCSS) has shown that the risk of death due to cardiovascular disease is eight times higher in survivors of an AC-treated neoplasm as compared to the general population [12, 13]. Considering the unfavorable prognosis of AC-induced cardiomyopathy [14], early identification of patients at risk by means of optimal cardiac function monitoring is essential both for the cardiologist and the oncologist, allowing timely implementation of personalized treatment regimens and possibly even prevention of cardiac dysfunction.

## 2. Chemotherapy-Induced Cardiotoxicity

### 2.1. General Toxicity

As stated, chemotherapy is the main method used for the treatment of pediatric leukemia. Although an effective treatment, one of its major drawbacks is the increased toxicity of the drugs being used, which sometimes counterbalances their therapeutic benefit [9–11, 15].

Anticancer drugs have general toxicity, explained by their action on cells with a high division rate, such as intestinal epithelia and hematopoietic cells. Thus, the most common side effects are bone marrow failure, digestive disorders (nausea, vomiting, and diarrhoea), and alopecia. These consequences cannot be avoided but in most cases resolve spontaneously when stopping the treatment. Specific toxicity is determined by the pharmacodynamics and pharmacokinetic particularities of each agent used.

In order to determine the life quality of cancer survivors, CCSS has monitored cancer treatment side effects on 14,357 survivors of pediatric malignancies treated between 1970 and 1986, with at least 5 treatment-free years at the moment of enrolment in the study. Analyzing the data, it has been found that survivors of childhood cancer have an eight times higher risk of developing chronic diseases as compared to their brothers or sisters. Also, more than a third will eventually develop a severe, potentially fatal condition [13]. In addition to the development of secondary malignancy, the most common side effects associated with the use of chemotherapies are cardiovascular disease, respiratory dysfunction, renal failure, infertility, psychosomatic development delay, and allergic reactions [13, 15, 16].

### 2.2. Cardiac Toxicity

The heart is a tissue with reduced regenerative capacity, so any extensive injury will cause irreversible damage. Although recent research has led to the development of even more effective antineoplastic agents, their effects on the myocardial tissue have not disappeared.

Cardiovascular side effects caused by chemotherapy are various, including arrhythmias and conduction disorders, heart failure (HF), acute coronary syndromes, myocarditis, and pericarditis. The most commonly encountered side effect is the alteration of left ventricular (LV) contractility, with the consequent decrease of its ejection fraction (LVEF).

In a simplified manner, postchemotherapy cardiotoxicity has been divided into two types: type I: caused by cardiomyocyte death, irreversible (most commonly associated with AC treatment), and type II: caused by myocardial dysfunction, frequently reversible (most commonly associated with Trastuzumab use) [17].

AC-induced cardiac dysfunction can also be divided into clinical and subclinical disease, by taking into account the presence or absence of clinical manifestations of congestive HF. In terms of subclinical changes, multiple definitions have been proposed, a widely accepted one being an alteration of the systolic function objectified by echocardiographic measurements or radionuclide angiography. Concerning the echocardiographic criteria, systolic dysfunction is considered to be present when LVEF is reduced by 10% for asymptomatic patients and 5% in symptomatic patients, or a decrease of LVEF below 50% [18].

### 2.3. Anthracycline-Induced Cardiac Toxicity

It is estimated that there are currently over 363,000 survivors of childhood cancer, with 60% of them believed to have been exposed to AC [19].

#### 2.3.1. Anthracyclines: The Mechanism of Toxicity

AC are a class of anticancer drugs, derived from Streptomyces Bacterium. They act at the nuclear level by DNA intercalation, topoisomerase 2*β* (TOP2*β*) inhibition, and production of reactive oxygen species (ROS), eventually triggering the pathways of cellular apoptosis [14, 20, 21]. Of all the classes of anticancer drugs used in the treatment of pediatric leukemia, AC are most known for their toxic effects on cardiac tissue [14, 18, 19]. These are effective antimitotics on many types of cancer, doxorubicin (DOX) being the most potent agent in this class, with the largest action spectrum. It is commonly used in oncology for both solid tumors and hematopoietic malignancies. However, the proven cardiac side effects of both DOX and daunorubicin limit their use [22]. More novel AC molecules such as Epirubicin and idarubicin and the structurally related molecule mitoxantrone have been proposed as less cardiotoxic variants of DOX. However, over the years, all types of AC have been shown to cause AC-induced cardiac toxicity [23].

**Figure 1:**
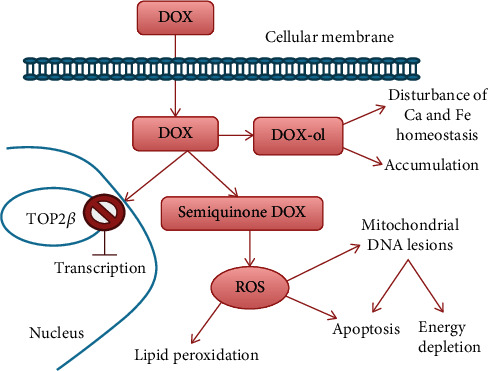
Doxorubicin (DOX): mechanism of action (DOX alcohol metabolites: DOX-ol).

The molecular mechanism for AC-induced cardiotoxicity (Figure 1) is complex and incompletely understood: cardiac toxicity is believed to be caused partly by the production of ROS and partly by the production of alcohol metabolites that accumulate in the myocytes [20].

Considering DOX, for example, the reduction of an electron from the quinone group leads to the formation of a semiquinonic radical, which will reduce the molecular oxygen to superoxide anion and hydrogen peroxide, both ROS. In this way, DOX causes oxidative stress and energy depletion at the cellular level, while also activating apoptotic pathways. Consequently, AC induce irreversible cardiomyocyte loss.

The second mechanism proposed, which explains the chronic, ongoing damage suffered by the myocardium, involves the conversion of AC to alcohol metabolites. These do not have the same oxidative potential as ROS but cause disturbances in calcium (Ca) and iron (Fe) cellular homeostasis, thereby affecting the contractile function. Also, being polar compounds, alcohols accumulate, which explains why cardiotoxicity risk increases proportionally to the total administered dose of AC [20, 21, 24].

Recent studies propose that TOP2*β* is involved in the development of increased oxidative stress following DOX treatment. AC bind to both TOP2*α*, which is overexpressed in cancerous cells, and TOP2*β*, expressed in adult mammalian cardiomyocytes. Studies showed that TOP2*β* cardiomyocyte knockout mice presented less impairment in cardiomyocyte function, while wild-type mice exhibited significant abnormalities in the p53 tumor suppressor gene, *β*-adrenergic signaling, and apoptotic pathways. [25]

The more the mechanisms of cardiotoxicity are understood, the easier it becomes to develop new cardioprotective treatment strategies, while also preserving the desired oncologic efficacy.

#### 2.3.2. Risk Factors for the Development of Anthracycline-Induced Cardiotoxicity

The incidence of cardiotoxicity after AC treatment is influenced by multiple factors, among the most important ones being the type of chemotherapy, the total given dose, and age at onset of therapy [26].

As stated, AC are one of the antineoplastic medications most frequently associated with long-term cardiac side effects following chemotherapy, the risk increasing proportionally to the total cumulative dose. At a total dose of less than 300 mg/m^2^, the risk of developing cardiotoxicity is considered to be 5%, increasing to 20% when the total dose exceeds 300 mg/m^2^ and to more than 35% at doses higher than 600 mg/m^2^ [27].

In the pediatric population, young age at diagnosis has been associated with an increased risk of subsequent cardiac damage. A study by Armstrong and Ross showed that childhood cancer survivors had twelve times higher risk of developing congestive HF following AC treatment in the following 3 years after treatment [28]. Also, another study showed that the incidence of AC-induced cardiac toxicity has risen up to 30% of the adult survivors of childhood cancer [29].

Other risk factors for AC-induced cardiac toxicity are preexisting cardiovascular risk factors such as diabetes, arterial hypertension, obesity, lung disease, or thyroid disease [30]. This is why, in the adult population, an increase in cardiotoxicity following AC treatment is noticed with age, as the elderly population already presents an increased prevalence of the above-mentioned additional cardiac risk factors.

#### 2.3.3. Clinical Manifestations: Prognosis

Cardiovascular complications caused by AC can be acute, chronic with early onset or chronic with late onset, depending on the time frame and reversibility of cardiac damage [9].

Acute toxicity occurs rarely during treatment, with an incidence lower than 1%, is dose-independent, and most often resolves shortly after treatment ends [31]. It may have various manifestations: myocarditis, pericarditis, and endocarditis. Acute HF during treatment is a rare but extremely serious side effect, as it requires immediate treatment termination [32]. Arrhythmias and hypotensive episodes are acute manifestations that occur more often during treatment but do not always require cessation of chemotherapy [9].

Chronic heart disease is a more common side effect of AC treatment. Depending on the onset of symptoms, cardiac damage may be subdivided into early-onset cardiotoxicity when symptoms occur within 1 year from finalizing the treatment or cardiotoxicity with late onset when symptoms occur after more than 1 year from finishing chemotherapy. The risk of developing cardiac toxicity increases proportionally to the treatment-free interval [33, 34]. Chronic cardiotoxicity manifests as a decrease in cardiac function leading to CHF. Unlike acute complications, chronic impairment is in most cases progressive [9, 10]. This toxicity has been shown to be dose-dependent and cumulative: initially, diastolic dysfunction occurs with a cumulative doxorubicin dose of 200 mg/m^2^, while systolic dysfunction occurs later, when the total dose exceeds 400-600 mg/m^2^, with individual variability [32, 33]. However, recent studies have shown that cardiac toxicity can occur even at doses previously considered “harmless” to cardiac tissue [35, 36].

Diastolic dysfunction is frequently asymptomatic, which is why careful cardiac monitoring of patients treated with anthracyclines is required even if they do not present any symptoms of cardiac disease [33]. Also, if diagnosed in the symptomatic phase, the prognosis and treatment response of AC-induced cardiomyopathy are poor with a 5-year survival rate below 50% [33, 37].

#### 2.3.4. Genetic Polymorphisms in Anthracycline Metabolism

A long-term follow-up of anthracycline-treated children has shown in some patients development of cardiac side effects at cumulative doses of less than 150 mg/m^2^, as well as a lack of toxic effects in some patients at over 600 mg/m^2^ [35]. This indicates the importance of individual variability in terms of pharmacodynamics and pharmacokinetics, most likely due to genetic polymorphisms.

In a recent study, the Children Oncology Group (COG) has shown that homozygous patients for the G allele of carbonyl reductase 3 (CBR3: an oxidoreductase involved in the reduction of carbonyl groups in alcohol groups, important in anthracycline metabolism) are at an increased risk of developing toxic cardiomyopathy even when low doses of AC are being used [38]. For these patients, it is considered that there is no risk-free dosage. Another study identified the polymorphisms of the SLC28A3 gene as an important modulator for the risk of developing AC-related cardiotoxicity [39].

A recent review on AC-related cardiotoxicity mechanisms and genomics in childhood cancer survivors revealed a total of 18 genes or genetic variants associated with AC-induced cardiac toxicity. These genes play roles in DNA damage pathways, oxidative stress response, iron metabolism, drug transport, and sarcomere function. Mostly, the ABCC, CBR3, and SLC28A3 genes have emerged in the majority of studies cited, emphasizing their important role in the development of AC-related heart disease [23].

These findings could facilitate, in the future, the implementation of targeted and personalized primary prophylactic strategies.

## 3. Monitoring Patients with Anthracycline

The risk of death by cardiovascular pathology is eight times greater in cancer survivors than the risk of tumor recurrence, especially in pediatric patients [9]. Cardiovascular damage dramatically reduces not only the duration but also the quality of life of these patients. Moreover, their response to standard cardiac treatments is often reduced and unsatisfactory.

Diagnosing cardiac toxicity at a stage where it is already symptomatic greatly limits the potential benefits of drug intervention, thus the importance of establishing a method that could aid in diagnosing AC-induced cardiomyopathy in its subclinical stages. This can be achieved by elaborating a specific follow-up protocol using the means we currently have, as well as developing new methods for early identification of patients at risk [27].

### 3.1. Echocardiography

Echocardiography is the most commonly used screening method for cardiac pathology, being an easily accessible, noninvasive, inexpensive, and fast method that allows real-time visualization of the heart.

Evaluation of the LVEF is essential for assessing heart function, being also a necessary tool in the diagnosis of AC-induced cardiomyopathy [27, 33]. Some studies also recommend the use of ventricular shortening fraction (SF) during the follow-up, with a SF lower than 30% indicating significant cardiac function impairment [40, 41].

However convenient, studies have shown that changes in LVEF or LVSF often show a rather irreversible alteration of heart function [32, 41]. Therefore, the European Society for Medical Oncology (ESMO) proposed the use of Doppler echocardiography for basal evaluation and periodic monitoring of cardiac function [42] as being a more sensitive method. What is more, the Pulse Wave Doppler (PWD) method has proven to be extremely useful, allowing for the assessment of flow velocities at a given point in real time. The PWD method records the magnitude of *E* and *A* waves at the level of the mitral valve, the ratio of which (*E*/*A*) is useful in diagnosing diastolic dysfunction.

Recently, Tissue Doppler Imaging (TDI) has become increasingly used, allowing diagnosis of cardiac impairment even in the stage of subclinical diastolic dysfunction. This method records myocardium motion velocities with the pulsed Doppler system set for low velocities. Using TDI, 3 wave patterns are recorded: the positive *S*′ wave (recorded in the systolic phase) and the negative *E*′ and *A*′ waves (recorded in the diastolic phase). Studies showed decreased rates of these waves in the AC-treated group versus the control group [43, 44]. These correlated with reduced systolic contraction and delayed relaxation, in apparently asymptomatic patients with normal LVEF and LVSF. This emphasizes the importance of using PWD and TDI for the timely detection of cardiac dysfunction.

Another method of identifying early cardiac damage is speckle tracking. This is an application of TDI, which calculates the strain and strain rate based on spatial differences in tissue velocity. Follow-up studies of oncological patients encourage evaluation of LV strain and global strain, the latter being preferred. However, these evaluations proved to be more useful in the immediate period following treatment and less in the long-term follow-up [45]. A recent study of 1,820 surviving, adult, pediatric cancer patients revealed a reduction in global longitudinal strain (GLS), as compared to normal values. However, the patients included in this study already had low LVEF, hypertension, or impaired glucose tolerance; therefore it was not possible to determine if GLS was reduced merely because of the former antineoplastic treatment [46].

Lastly, echocardiography greatly depends on the operator, the results being greatly influenced by their knowledge. All things considered, the ideal imaging method of cardiac function evaluation for these patients is still to be determined.

### 3.2. Electrocardiogram (ECG)

ECG is a noninvasive method used to evaluate cardiac conductive tissue, allowing identification of arrhythmias, conduction anomalies, and cardiac ischemia. There are studies that correlated a prolonged QT interval in oncological patients with the increased possibility of later developing a cardiac pathology [47]. Acute DOX toxicity includes supraventricular tachycardia, ventricular ectopy, myopericarditis, cardiomyopathy, and death. However rare, these manifestations are life-threatening; thus, ECG examination is required in the follow-up protocol of these patients.

### 3.3. Biomarkers

In recent years, interest in the use of biological markers has increased due to the need to easily identify patients at risk of developing chemotherapy-related cardiac toxicity.

#### 3.3.1. C-Reactive Protein (CRP)

CRP is an acute-phase protein synthesized in the liver. In patients with heart disease, high levels of CRP signal a proinflammatory status and correlate with the HF severity, indicating a negative prognosis. Also, highly sensitive CRP (hs-CRP) is a reliable indicator for the risk of an acute cardiovascular event, values higher than 3 mg/l being associated with an increased risk [27].

#### 3.3.2. Tumor Necrosis Factor Alpha (TNF*α*)

TNF*α*, Interleukin- (IL-) 1, IL-6, and IL-18 are proinflammatory cytokines. IL-6 induces myocardial hypertrophy, while TNF*α* activates matrix metalloproteinases, inducing LV dilatation. The two cytokines have been used as predictive markers for the development of HF in elderly patients [48].

#### 3.3.3. Markers of Oxidative Stress

Since it is difficult to assess cellular oxidative stress, it was attempted to estimate it using indirect markers such as oxidized low-density lipoproteins, malondialdehyde, and myeloperoxidase. In animal models, administration of doxorubicin increased both the activity of myeloperoxidase and lipid peroxidation [49].

#### 3.3.4. Natriuretic Peptides

Brain natriuretic peptide (BNP) and N-terminal prohormone BNP (NT-proBNP) are two extremely useful markers in cardiac function assessment. These are synthesized in the myocyte in response to increased cardiac wall pressure. BNP produces vasodilatation, increases diuresis and natriuresis, and reduces sympathetic nervous system activity and renin-angiotensin-aldosterone system activation. They are used to diagnose HF (at a level above 400 pg/ml), to stratify the patients in risk groups, and also in their long-term follow-up [50].

Recently, the utility of these markers has been demonstrated for identifying patients at risk of developing cardiotoxicity. In a study by Sandri et al., 52 patients who received high-dose chemotherapy were evaluated. NT-proBNP values were determined at onset and at the end of treatment, as well as at 12, 24, 36, and 72 hours after. The values of 33% of patients remained elevated and 72 hours posttreatment. This group demonstrated a decrease in LV diastolic index and a reduction in LVEF from 62% to 45% in the year following treatment [51].

#### 3.3.5. Markers of Myocardial Injury

Cardiac Troponins (cTn) T and I are myofibrillar proteins that have demonstrated increased sensitivity and specificity as markers of myocardial injury. Several studies have shown increased cTnT levels in the early stages of AC therapy [52]. This increase was correlated in some studies, with a marked reduction in the diastolic function of LV [53, 54]. In a study on patients with breast cancer treated with Trastuzumab, cTnI has proven to be an important predictor of cardiotoxicity as well as a negative prognostic factor regarding cardiac function recovery [18]. Following these studies, in 2010, Cardinale and Sandri proposed cTn levels to be used in cardiac risk assessment for both standard anticancer treatments and new biological therapy [54]. Also, in a study of 18 pediatric patients diagnosed with non-Hodgkin's lymphoma, Blaes et al. showed that patients with elevated cTn at the beginning of treatment had an increased incidence of systolic dysfunction [55].

A recent review analyzing over 20 studies regarding cTn use as a biomarker of cardiotoxicity in patients treated with AC for breast cancer concluded that the main evidence up until today is that low cTn levels during treatment correlate with a better long-term prognosis regarding heart function [56].

### 3.4. Monitoring during Treatment

Monitoring during treatment has a role of identifying potential cardiac damage as soon as possible, thus allowing therapeutic interventions and treatment modification. The goal is to reduce the risk of developing long-term cardiac complications [11]. At the same time, it should be taken into account not to reduce treatment's efficacy, which would eliminate the benefit created by reducing cardiotoxicity.

A recent study on pediatric leukemia has shown that myocardial tissue is affected even before chemotherapy begins, as seen from the correlation determined between the white blood cell count at diagnosis and NT-proBNP values. This might be partially explained by myocardial infiltration with cancer cells. However, preexisting cardiac suffering highlights even more the need for a timely, rigorous, ongoing cardiac function evaluation [57].

In order to be effective, Steinherz et al. emphasize the importance of conducting an ECG and echocardiography prior to the beginning of treatment [58]. Subsequently, most guidelines recommend an ultrasound after half the total cumulative dose of doxorubicin is given, followed by an echocardiographic examination before each of the following doses [58]. It has been proposed that at a decrease in LVEF below 50% or more than 10% during treatment, chemotherapy should be discontinued. This is based on the fact that the identified systolic dysfunction appears most likely following an extensive myocardial injury [27]. However, a lack of reduction in LVEF during treatment does not rule out the possibility of late cardiac toxicity [27, 33, 43, 59].

### 3.5. Long-Term Monitoring

Lifetime screening for cardiac damage is indicated following antineoplastic treatment, especially in patients treated with AC or those who have received radiation therapy to the chest.

In the first year following treatment, ultrasound screening is currently recommended at 3, 6, and 12 months [26]. COG provides a detailed guide on the frequency of posttreatment monitoring, based on age at exposure to AC, the total dose received, and the association with thoracic irradiation. For a universal approach, they propose converting all doses of AC to isotoxic doses of doxorubicin [38].

Another important aspect is the screening for cardiovascular risk factors: sedentary lifestyle, tobacco use, family history of premature coronary heart disease (less than 55 years in men and 65 years in women, respectively), lipid profile, basal blood glucose, and blood pressure (BP). Cancer patients are generally considered at risk for development of cardiovascular pathology, so adding any other two cardiac risk factors leads to the inclusion of these patients in a high-risk group. Thus, according to the American Heart Association, for cancer survivors, the target body mass index (BMI), BP, LDL, and glucose levels change: BMI < 90th percentile, BP < 95th percentile, LDL < 130 mg/dl, and basal blood glucose < 100 mg/dl [60].

## 4. Therapeutic Outlook for Anthracycline-Induced Heart Failure

First of all, in order to decrease the likelihood of AC-induced cardiac disease, the administration recommendations have been modified. The maximum total cumulative dose recommended nowadays being 400-550 mg/m^2^ DOX and 900 mg/m^2^ Epirubicin. Anyhow, one must keep in mind that up until now no dose of AC has been considered cardiac risk-free, so the ongoing evaluation of these patients is mandatory regardless of the received dose. Also, a slow DOX infusion has proven to diminish the cardiotoxic effect of AC use, by lowering its maximum plasma levels, a parameter which, in turn, determines the amount of drug entering the myocardial tissue [61]. However, Lipshultz et al. conducted a study on 102 children treated for ALL, who received doxorubicin in a randomized fashion, either in a continuous regimen (over 48 hours) or by bolus (15 minutes). A cardiac follow-up, with a median of 8 years, showed no significant difference in cardiac function between the two groups, concluding that, in children, continuous infusion shows no benefit over bolus administration [62].

The use of liposomal drug formulations has been widely debated and studied. Liposomal DOX has the advantage of a limited diffusion through the myocardial tissue, due to their size (too big to cross the endothelial junction of healthy tissues) with preserved antitumor efficiency (leaky, irregular tumor vasculature) [63]. There are many successful animal studies done on solid tumors, which show not only the preserved desired antitumor effect with minimal cardiac toxicity but also, in some cases, liposomal formulations actually exposing tumor cells to higher amounts of AC [64]. However good the results are, there are few randomized clinical trials on liposomal-coated AC, thus the limited clinical indications so far being metastatic breast cancer, advanced ovarian cancer, multiple myeloma, and AIDS-related sarcoma [65]. Until further studies emerge, liposomal formulations are not yet an alternative for children with leukemia.

Regarding preventive treatment, cardioprotective drugs such as dexrazoxane, angiotensin-conversion enzyme inhibitors, and beta-blockers have been tested [21, 66]. Dexrazoxane, an iron chelating agent, has long been considered the first-line prophylactic therapy for chemotherapy-induced cardiac toxicity, being the only drug currently approved by the US FDA for the prevention of AC-induced HF. It has also been proven to be efficient in children with leukemia. Lipshultz et al. demonstrated in a randomized controlled trial of 205 children the protective effect of dexrazoxane on cardiac function as means of LV structure and function, with no adverse effect on relapse risk, frequency of secondary malignancy, or survival [67]. Another randomized controlled trial, from the Pediatric Oncology Group, has shown that, although the 5-year survival rate did not differ between the group that received dexrazoxane and the group without it, measurements of the SF, LV wall thickness, and thickness-to-dimension ratio were worse in patients who did not receive dexrazoxane [68]. However, in 2007, a controversial study claimed that dexrazoxane use could increase the risk of secondary malignancies, especially AML [69]. No further studies have supported this theory so far [70]. What is more, after previously allowing dexrazoxane to be used only in women treated for breast cancer, the EMA changed its decision and now supports its administration to pediatric patients who are likely to be treated with high cumulative doses of anthracyclines (>300 mg/m^2^ of doxorubicin) [71, 72].

Beta-blocker use is encouraged in a recent review on their role in the prevention of AC-induced cardiotoxicity, due to their important cardioprotective action. Carvedilol seems to be the most studied drug from this class; however, its dosing regimens and optimal timeline of administration in oncologic patients still need to be established [73]. A small study of 25 patients demonstrated that Carvedilol administration started before initiating AC therapy improved LVEF and the value of the *E*/*A* ratio compared to the placebo group [74]. Similar studies were also performed using Enalapril, Spironolactone, Metoprolol, and Candesartan, all with encouraging results in the prevention of postchemotherapy cardiotoxicity [75–78]. Another study conducted on 473 cancer patients presenting with elevated cTn following various cytostatic regimens demonstrated that Enalapril administration for over a year resulted in a lower incidence of LV dysfunction than in the placebo group [79].

For patients who have already developed HF secondary to cytostatic treatments, there are limited studies regarding the appropriate therapeutic approach. For now, HF is to be treated according to the current guidelines, although treatment response is poorer than in the “classical” HF patient population.

## 5. Conclusion

Taking all the above-mentioned aspects into account, it is obvious that cardiotoxicity following AC treatment is a current issue for both the oncologist and the cardiologist. The pediatric population represents an even bigger challenge, because of the various stages of development in which children receive chemotherapy, being very difficult to establish specific monitoring and treatment protocols. There are many questions unanswered in cardiooncology, thus the need and development of a separate medical specialty dealing with this intricate problem. All things considered, careful and systematic monitoring, as well as timely intervention, proves to be crucial to the long-term prognosis and quality of life for these patients.

## Data Availability

The data supporting this review are from previously reported studies and datasets, which have been cited.
